# Disruption of Tumor Suppressors HNF4α/HNF1α Causes Tumorigenesis in Liver

**DOI:** 10.3390/cancers13215357

**Published:** 2021-10-26

**Authors:** Aamir Salam Teeli, Kamila Łuczyńska, Effi Haque, Mohmmad Abrar Gayas, Dawid Winiarczyk, Hiroaki Taniguchi

**Affiliations:** 1 Institute of Genetics and Animal Biotechnology of the Polish Academy of Sciences, 05-552 Jastrzebiec, Poland; teeliaamir7@gmail.com (A.S.T.); k.luczynska@igbzpan.pl (K.Ł.); e.haque@igbzpan.pl (E.H.); d.winiarczyk@igbzpan.pl (D.W.); 2 Department of Surgery and Radiology, Faculty of Veterinary Sciences and Animal Husbandry, SKUAST-K, Jammu 19000, India; abrargayas@gmail.com

**Keywords:** hepatocellular carcinoma, HNF4α, HNF1α, inflammation, β-catenin, EMT

## Abstract

**Simple Summary:**

Liver cancer is one of the deadliest human cancers. High-throughput analysis of cancer cell genomes has established that hotspot mutations in HNF4α and HNF1α occur in a variety of human cancers, including liver cancer. Here, we review recent findings pertaining to role of HNF1α and HNF4α in liver cancer, and their possible targeting for liver cancer treatment.

**Abstract:**

The hepatocyte nuclear factor-4α (HNF4α) and hepatocyte nuclear factor-1α (HNF1α) are transcription factors that influence the development and maintenance of homeostasis in a variety of tissues, including the liver. As such, disruptions in their transcriptional networks can herald a number of pathologies, such as tumorigenesis. Largely considered tumor suppressants in liver cancer, these transcription factors regulate key events of inflammation, epithelial–mesenchymal transition, metabolic reprogramming, and the differentiation status of the cell. High-throughput analysis of cancer cell genomes has identified a number of hotspot mutations in HNF1α and HNF4α in liver cancer. Such results also showcase HNF1α and HNF4α as important therapeutic targets helping us step into the era of personalized medicine. In this review, we update current findings on the roles of HNF1α and HNF4α in liver cancer development and progression. It covers the molecular mechanisms of HNF1α and HNF4α dysregulation and also highlights the potential of HNF4α as a therapeutic target in liver cancer.

## 1. Introduction

Transcriptional factors (TFs) play a pivotal role in normal cellular physiology, and their aberrant expression is linked to a myriad of diseases [1]. Mutations in TFs and TF-binding sites underlie many human diseases, including cancer. The hepatocyte nuclear factor 4α (HNF4α, NR2A1, gene symbol HNF4A) and hepatocyte Nuclear Factor 1α (HNF1α) TFs influence the development as well as the maintenance of normal homeostasis in a variety of tissues including the liver, kidney, and small intestine [2,3]. The HNF family of TFs is implicated in mature onset diabetes of the young (MODY), and mutations in the HNF4α, HNF1α, and HNF1β genes cause MODY, a form of non-insulin-dependent diabetes mellitus [4,5,6]. HNF4α is a member of the ligand-dependent NR2A nuclear receptor superfamily of TFs [7,8]. HNF4α is highly expressed in the liver, in which about 60% of the actively transcribed genes have a binding site for HNF4α [8,9]. It is crucial during embryonic development and liver organogenesis, as manifested by embryonic lethality in HNF4α-knockout mouse embryos [10,11]. The HNF4α protein contains six distinct domains: an N-terminal activation domain (activation function-2), a DNA-binding domain (DBD) containing two zinc finger motifs, a presumed ligand-binding domain, a C-terminal homodimerization and activation domain, and a repressor domain [2,12]. The expression of HNF4α is driven by two distinct promoters, P1 and P2, which result in 12 different variants (HNF4α 1-12) with an organ-specific expression [13]. The zinc finger region is essential for the binding of HNF4α to its target promoters and the recruitment of coactivators and corepressors, such as p300, SRC-1, and p160, to the target site [14,15,16,17]. HNF4α is a master regulator of cell fate decisions and maintains the differentiated state of many cell types, including hepatocytes [11]. While HNF4α has been well studied in the development of MODY, its role in tumorigenesis is not fully understood. Recently, many studies have proved its role as a tumor suppressant across different cancer types, including liver cancer [18,19], renal cell cancer [20], pancreatic cancer [21], and colorectal cancer [22,23]. Many studies have focused on liver cancer because of the pivotal role of HNF4α in the normal physiology and development of the liver. HNF4α plays a role throughout the initiation, malignant transformation, and metastasis in liver cancer development by regulating the key events of inflammation [24,25], epithelial–mesenchymal transition (EMT) [26,27], and the differentiation status of the cells [11,28]. Another liver-enriched TF, HNF1α acts synergistically with HNF4α to regulate gene expression in a variety of tissues and organs, including the liver, pancreas, and kidney [16,29]. HNF1α is present in embryonic tissues and affects cellular differentiation and organ development [30]. HNF1α interacts with target DNA as a homodimer or heterodimer with HNF1β to regulate glucose metabolism, lipid transport, and detoxification [4,31,32,33]. HNF1α is a member of the homeobox family of proteins, having a tripartite domain structure. Structurally, HNF1α has an N-terminal dimerization domain, a central DBD, and a C-terminal transactivation domain. The DBD is composed of a POU (Pit1, Oct1, and Unc1)-homeodomain (POU_H_) and POU-specific (POU_S_) parts and is not the prototype of homeobox proteins because of a unique 21-amino acid insertion in the POU_H_ part, which interacts with the POU_S_ to stabilize the interface for efficient transcriptional activity [31,34]. Recent data from many studies have established HNF1α as a tumor suppressor [35,36,37,38]. The expression of the HNF4α gene is mainly upregulated by transcription factor HNF1α, which binds to the HNF4α promoter region together with HNF6 [39]. HNF4α also occupies the HNF1α promoter region and upregulates its expression as positive feedback, but HNF1α inhibits its own gene transcription by protein–protein interaction with HNF4α as negative feedback [40]. HNF4α and HNF1α together form a network of transacting factors regulating the expression of each other and multiple liver-specific genes [41,42,43]. HNF4α and HNF1α act together as a heterodimer at many target genes and reciprocally regulate each other’s expression through DNA-binding-dependent and independent (protein–protein interaction) mechanisms. HNF4α facilitates the recruitment of p300 and enhances HNF1α-mediated transcription activation [16]. Several cancer genome consortia, like the International Cancer Genome Consortium (ICGC), study the genomic changes in different types of cancers and publish in-depth lists of somatic mutations in cancer. The analysis of these somatic mutations can reveal previously unidentified pathways in carcinogenesis and new targets of cancer treatment. The ICGC data analysis revealed that, alongside top mutated genes, HNFs are also mutated in liver cancer and may be implicated in carcinogenesis. Though HNFs are well-established tumor suppressants in the liver, their functional importance is yet to be elucidated.

## 2. The Roles of HNF4α and HNF1α in Liver Cancer Development

### 2.1. HNF4α and HNF1α as a Link between Inflammation and Cancer

**Figure 1:**
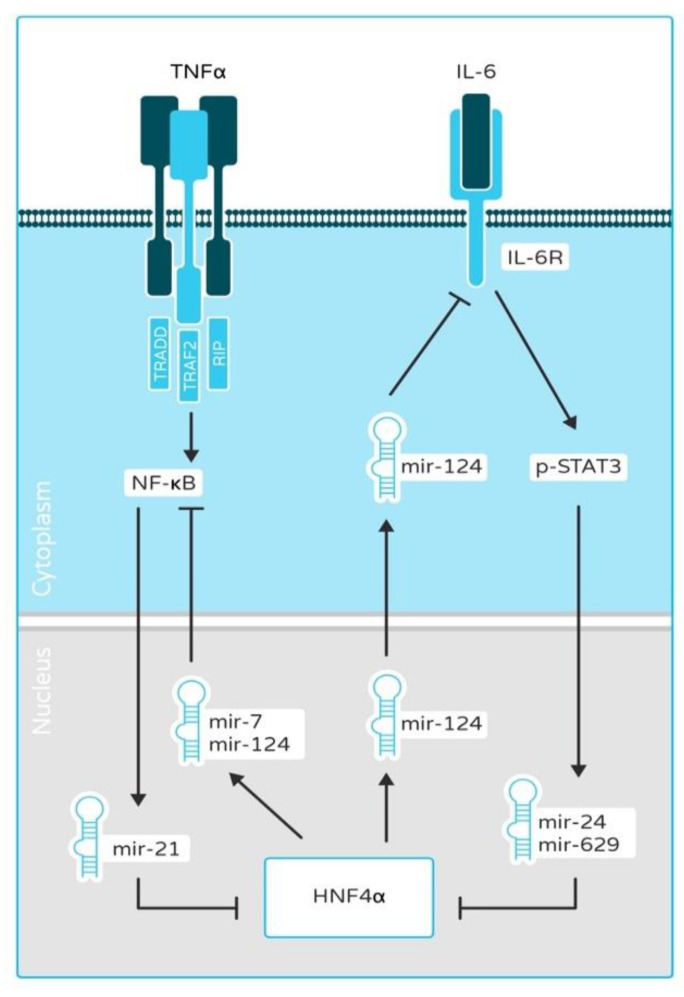
Role of aberrant HNF4α-inflammatory signaling in cancer. A regulatory pathway involving microRNAs and HNF4α links inflammatory responses (TNFα, IL-6) to hepatocyte transformation and epithelial-mesenchymal transition (EMT). After induction by stimuli, NF-κB and IL-6 inhibit the expression of HNF4α by inducing the expression of miR-21, and miR-24, miR-629, respectively. High HNF4α expression inhibits the NF-κB and IL-6 pathways by upregulating the expression of miR-7, miR-124 microRNAs. Aberrant HNF4α-related NF-κB, STAT3 and IL-6 signaling pathway is involved in tumorigenesis and the EMT process.

Inflammation is an adaptive immune response to infectious pathogens and tissue injury, characterized by immune cell recruitment and the release of cytokines to ward off the infection or harmful impetus, restore damaged tissues, and maintain homeostasis. However, under the conditions of persistent inflammatory stimuli or the dysfunction of regulatory mechanisms, non-resolving inflammation occurs, which sets the stage for autoimmunity and cancer development [44]. The preponderance of evidence suggests a clear link between inflammation and its protumorigenic role, which is the consequence of its role in tissue regeneration and repair. Up to 20% of cancers have been linked to chronic inflammation or infections [45,46,47]. Liver cancer, gastric cancer, and colorectal cancer (CRC) may differ in their etiology and pathogenic mechanisms, but inflammation is a common factor. A study by Hatziapostolou et al. established HNF4α as a candidate linking inflammation and hepatocarcinogenesis through microRNA regulation [25] (Figure 1). The IL6-STAT3 pathway is implicated in driving liver tumorigenesis by inducing the expression of oncogenic microRNAs, miR-24 and miR-629. These oncomiRs, in turn, inhibit HNF4α expression and result in hepatocyte neoplastic transformation. A target of HNF4α, miR-124 is a well-established inhibitor of the IL6-STAT3 pathway, directly targeting the IL6R [25]. The perturbation of the HNF4α-miRNA inflammatory circuit may result in hepatocarcinogenesis. The anti-inflammatory role of HNF4α is also evidenced by its protective action against inflammatory bowel disease (IBD) and ulcerative colitis [23]. Uncovering the role of HNF4α in inflammation is a crucial area of research and can offer new therapeutic options for inflammation-driven cancers.

The constitutive activation of the nuclear factor kappa B (NF-kB) signaling pathway is vital in establishing an inflammatory environment that is conducive to cellular transformation and neoplasia. NF-kB regulates the expression of a myriad of genes involved in cellular proliferation, apoptosis, survival, and tumorigenesis [48]. One of the predisposing factors for liver cancer is fibrosis [49]. An inflammatory microenvironment facilitates the onset of fibrosis and, ultimately, liver cirrhosis, which sets the stage for hepatocarcinogenesis [50]. The activated hepatic stellate cells (HSCs) are the cells implicated in inducing fibrosis; they do so by inhibiting HNF1α expression in hepatocytes through IL-6- and TNFα-induced expression of miR-21 and miR-146a in the hepatocytes and thus promoting tumor development [38]. Highly expressed in epithelial and hematopoietic cells, SHP-1/PTPN6 plays a prominent anti-inflammatory role by negatively regulating the NF-kB, ERK, STAT, JNK, and JAK2 pathways [51]. The SHP-1 promoter contains binding sites for HNF1α, and its expression is transcriptionally regulated by HNF1α in hepatocytes [38]. HNF1α negatively regulates the NF-kB pathway by inducing the expression of miR-194. In hepatocellular carcinoma (HCC), TNFα-induced NF-kB signaling downregulates HNF1α, thereby inhibiting miR-194, and results in tumorigenesis [52]. In summary, HNFs are vital in regulating inflammatory pathways and thus may be of importance in inflammation-associated cancer.

### 2.2. HNF4α and HNF1α in Epithelial-to-Mesenchymal Transition (EMT) and Liver Cancer Metastasis

**Figure 2:**
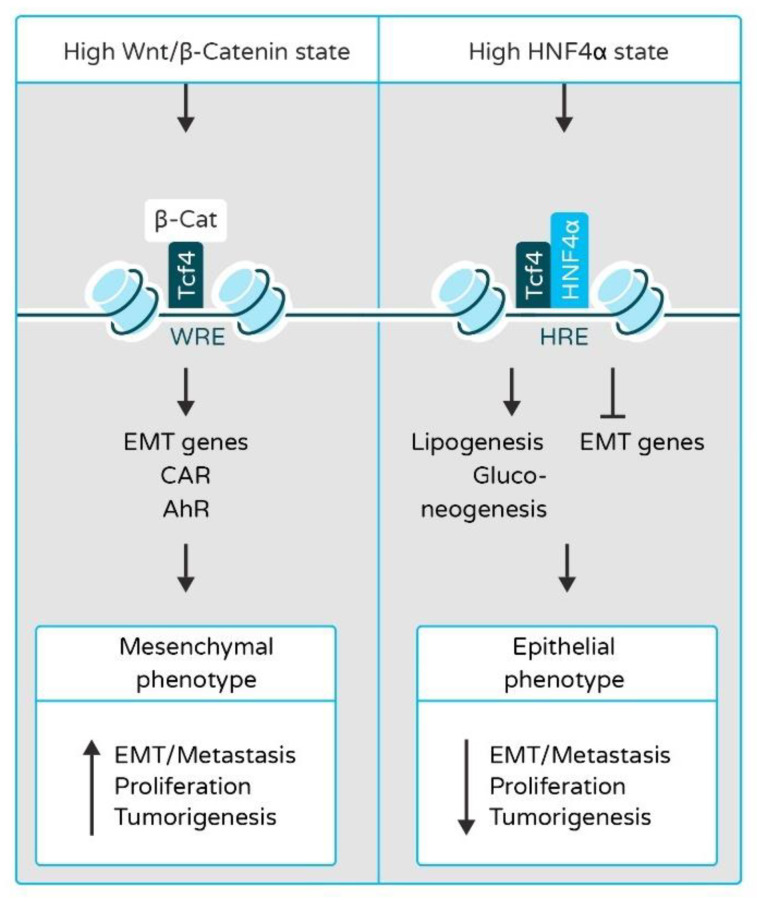
Interactions among β-catenin, HNF4α, and T-cell factor 4 (Tcf4) in hepatocytes determines the progression of hepatocarcinogenesis. In high β-catenin state, β-catenin binds to Tcf4 and this complex binds to Wnt response elements (WREs) in promoters of target genes in hepatocytes (like constitutive androstane receptor, CAR, and aryl hydrocarbon receptor, AhR) and competitively inhibits the expression of HNF4α and HNF4α responsive genes, thus promoting EMT and tumorigenesis (Left panel). In high HNF4α state, HNF4α binds to HNF4α response elements (HREs) in promoters of target genes to induce their expression and inhibits epithelial–mesenchymal transition (EMT) and tumorigenesis. Furthermore, HNF4α inhibits β-catenin transcription and prevents β-catenin/TCF complex binding to target gene promoters (Right panel).

The EMT-controlling TFs are well recognized to play a role in cancer development from tumor initiation to tumor dissemination [53]. The malignant cells must undergo EMT to acquire metastatic potential. EMT is tightly regulated by Wnt/β-catenin signaling and TFs such as SNAIL, SLUG, AXIN2, and TWIST. Under normal conditions, β-catenin is bound by a destruction complex containing Axin, APC, and GSSK3, which leads to β-catenin phosphorylation and destruction [54]; however, owing to the constitutively active Wnt signaling in tumor cells, β-catenin is stabilized [55]. It consequently forms a complex with T-cell factor (TCF) in the nucleus and upregulates the expression of EMT inducers, which leads to the change of polarity of epithelial cells as they transition to the mesenchymal cell type [56] (Figure 2). HNF4α is responsible for maintaining the differentiated phenotype of adult hepatocytes by inducing liver-specific genes and acting as a competitive inhibitor of the Wnt/β-catenin pathway and a direct inhibitor of EMT-inducing TFs [26,27,57,58] (Figure 2). HNF4α not only inhibits the nuclear translocation of β-catenin but also causes its trafficking to the plasma membrane, where it influences cell adhesion and connects cadherins to α-catenin and the cytoskeleton [26]. HNF4α also inhibits hepatocyte dedifferentiation, proliferation, migration, and malignant transformation by upregulating miR-194 and miR-192, which are negative regulators of cell adhesion and migration, activated leukocyte cell adhesion molecule (Alcam), tumorigenesis and tumor progression (Rap2B and epiregulin (Ereg)), and EMT-related genes (moesin (Msn)) [59]. HNF1α also plays a major role in maintaining the differentiated and epithelial phenotype of hepatocytes. The silencing of HNF1α in hepatocellular carcinoma cells by siRNA impairs their epithelial phenotype, characterized by the decreased expression of epithelial markers, such as E-cadherin, and the increased expression of mesenchymal markers, such as vimentin, and TFs involved in EMT like Snail1 and Snail2 [60,61,62].

### 2.3. HNF4α and HNF1α in Metabolism and Liver Cancer

The hallmark feature of tumor cells is metabolic reprogramming characterized by high glycolysis and lipogenesis (the Warburg effect) to cope with poor oxygenation and nutrient scarcity in the tumor microenvironment [63]. The metabolic adaptations in the tumor cells are the downstream effects of oncogenic signaling. Multiple lines of evidence suggest that disrupting the lipid metabolic pathways reduces tumor growth and metastasis and can offer new avenues for cancer treatment [64]. The peroxisome proliferator-activated receptor gamma (PPARG) is a master regulator of lipid uptake, synthesis, and storage in adipose tissue [65]. In hepatocellular adenoma (HCA) and a subset of HCC, the expression and activity of PPARG is significantly increased, affecting metabolic rearrangements and liver tumorigenesis via the transcriptional regulation of hexokinase 2 (HK2) and M2 pyruvate kinase (PKM2) [36,66]. In a whole-body mouse mutant of HNF1α, there was a significant fold induction of hepatic PPARG transcript and protein expression, suggesting a functional interaction between these two TFs. The same study established that PPARG is under the negative transcriptional regulation of HNF1α [36].

Non-alcoholic fatty liver disease (NAFLD) and its progressive subtype non-alcoholic steatohepatitis (NASH) are recognized as emerging causes of HCC. NAFLD is a group of diseases characterized by the deposition of fat in the hepatocytes, with obesity, diabetes, and insulin resistance identified as common risk factors. NAFLD starts as simple steatosis that progresses to NASH because of the actions of inflammatory mediators, reactive oxygen species (ROS), and intestinal microbiota [67,68,69,70]. The damage-associated molecular patterns (DAMPs; for example, HMGB1, cholesterol esters) and microbe-associated molecular patterns (MAMPs; for example, LPS) from the intestinal microbiota and damaged hepatocytes lead to the activation of toll-like receptor (TLR) signaling in resident immune cells and hepatocytes, which may support the development of NASH [68]. Defining the pathogenesis of NAFLD and NASH is the subject of intense investigation, and many studies have established a pivotal role for HNF4α and HNF1α in NAFLD-NASH. Whole-body knockout *Hnf1α* mice suffered from hyperglycemia, hypercholesterolemia, and fatty liver, indicating the essential role of HNF1α in liver function and fat metabolism [71,72]. Moreover, Ni et al. studied the effects of the hepatocyte-specific deletion of *Hnf1α* using the Cre-LoxP method and demonstrated a critical link between HNF1α and the development of NAFLD-HCC [73]. The *Hnf1α* knockout (KO) mice developed fatty liver, NASH, and liver tumors characterized by collagen deposition and fibrosis with no evidence of cirrhosis. Immunohistochemical staining of the tumor nodules revealed intense expression of glypican-3 (Gpc3), a diagnostic marker for HCC. Furthermore, the inflammatory and pro-proliferative pathways were highly active in the livers of *Hnf1α* KO mice, as evidenced by the increased expression of TNFα, TGFb1, IL-6, phosphorylation of NF-kB subunit p65 and Akt [73].

Liver-specific *Hnf4α* KO mice display increased plasma bile acid levels and reduced plasma levels of triglycerides and cholesterol owing to impaired, very low-density lipoprotein (VLDL) secretion by the liver [74]. In mice, the short hairpin RNA (shRNA)-mediated knockdown of hepatic Hnf4α leads to the dysregulation of genes involved in lipid metabolism, such as VLDL secretion (Mtp, ApoB), de novo cholesterol biosynthesis (Hmgcr, Hmgcs, Srebp-2), cholesterol catabolism (Cyp7a1, Cyp8b1), cholesterol esterification (Acat2, Lcat), and cholesterol uptake (Ldlr, SR-BI), which eventually leads to the development of fatty liver [75]. The expression and protein levels of HNF4α and mRNA levels of HNF4α target genes are drastically reduced in NASH patients [76]. The roles of microRNAs have been indicated in NAFLD and NASH [77], and, in particular, miR-34a is highly expressed during common metabolic stress (diabetes, HFD feeding, and NASH). Furthermore, miR-34a inhibits HNF4α expression via binding to the 3′-UTR and influences the regulation of hepatic and plasma lipid metabolism. During metabolic stress, hepatic miR-34a negatively regulates HNF4α, and the perturbation of this circuit may play an important role in NASH development [76].

### 2.4. HNF4α and HNF1α Mutations in Liver Cancer

**Figure 3:**
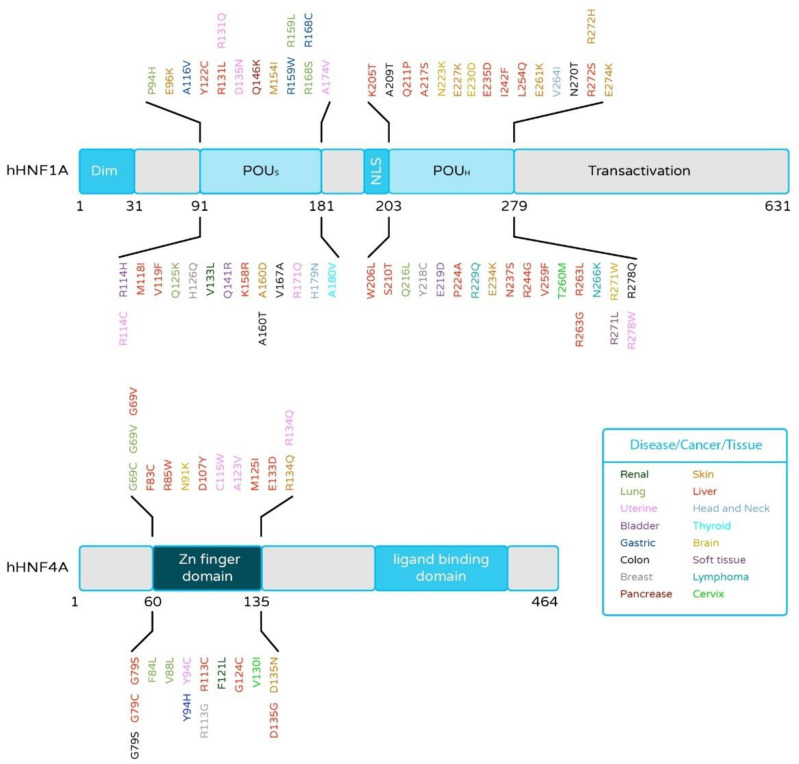
HNF1α and HNF4α mutations are found in their DNA-binding domain (DBD) in several cancers identified by the International Cancer Genome Consortium (ICGC). The amino acid positions of the identified mutations of HNF1α and HNF4α are shown, and the color coding indicates the type of cancer tissue in which the mutations were reported.

Cancers arise from the accretion of mutations in critical genes that alter the normal plans of cell proliferation, differentiation, and death [78]. The ICGC data analysis revealed relatively rare gene mutations, which may play crucial roles in tumor formation (ICGC; https://dcc.icgc.org/; accessed on 15 July 2021). High-throughput analysis of cancer cell genomes has established that hotspot mutations in HNF4α and HNF1α occur in a variety of human cancers, including liver [30,79,80,81,82,83], renal [84], colorectal [85], and pancreatic carcinomas (ICGC Database) (Figure 3). Notably, most of the HNF4α mutations are located in the Zn-finger region or the ligand-binding domain of HNF4α, which is well conserved among various species. Mutations in such evolutionarily conserved elements suggest a strong effect on the protein function, though further studies are necessary. Our group identified, for the first time, functional HNF4α mutations in liver cancers. Notably, HNF4α DNA-binding ability and transcriptional activity were lost in the tested mutations [14]. The study suggested that HNF4α loss-of-function mutations cause the reduction of HNF4α target gene expression, and, in turn, this may induce hepatic tumorigenesis or tumor growth. Additionally, the identification of HNF4α and HNF1α as mutated targets in cancer suggests that systematic searches through cancer cell genomes for somatic mutations will ultimately provide a full picture of the pathophysiology and therapeutic opportunities for underlying human oncogenesis. Therefore, the analysis of these somatic mutations may reveal new pathways involved in carcinogenesis and new targets of cancer treatment.

The mutations of HNF1α are well established in HCA characterized by hepatic steatosis due to increased fatty acid synthesis and decreased expression of liver fatty acid-binding protein (LFABP). The metabolic consequences of biallelic mutations of HNF1α are dysregulated glycolysis, gluconeogenesis, and lipogenesis [37,81]. The neoplastic consequence is the increased activity of the ErbB2 receptor tyrosine kinase, which activates the mTOR signaling pathway with proproliferative and anti-apoptotic effects [86,87]. HCAs are characterized by the lack of activation of β-catenin and CTNNB1 (Catenin Beta-1) mutations, and defective HNF1α signaling [88]. A study by Hechtman et al. reported two mutations, HNF1α E32 and L214Q, in HCC [82]. Ding et al. reported HNF1α Q511L somatic mutations in HCC defined by reduced transactivation activity and impaired nuclear localization of HNF1α [83]. The somatic mutations of HNF1α in HCC attenuate the tumor suppressor function of HNF1α and may play a role in HCC development through a distinct pathway independent of HCC with β-catenin mutations [88].

### 2.5. Phenotypic Consequences of Downregulated Expression of HNF4α and HNF1α in Liver Cancer

Both HNF1α and HNF4α act as tumor suppressors, and they reduce expression of factors that can trigger tumor development in several types of cells. The knockout of HNF4α in the adult mouse liver induces a robust proliferative response in normal hepatocytes [19]. Moreover, the heterozygous mutation of HNF4α resulted in the loss of transcriptional activation of target genes, APOB and HNF1α [89]. Additionally, the knockdown of HNF4α led to substantial reductions in HNF1α and APOB, and RNA-seq data from liver cancer patients also showed that the expression of HNF4α, HNF1α, and APOB mRNA are significantly correlated [14]. A recent study found that liver specific *HNF4α* knockout (H4LivKO) mice had enlarged livers with evident steatosis [90]. Gene expression analysis confirmed that Apolipoprotein M (apoM) was downregulated in H4LivKO mice; however, both Hnf4γ and PPARγ were upregulated in the liver [90], suggesting the effect of HNF4α ablation on lipid metabolism and liver function. The role of HNF4α in liver regeneration (LR) after partial hepatectomy (PH) was indicated by the decreased expression of HNF4α target genes after PH; the phosphorylation of HNF4α by Src kinase may be one of the mechanisms through which HNF4α exits the nucleus and stops transcriptional regulation after PH [91]. Moreover, proliferating cell nuclear antigen (PCNA)-positive nuclei were significantly higher in HNF4α-KO mice throughout the 7-day course post-PH, which indicated increased hepatocyte proliferation in HNF4α-KO livers throughout LR [91]. Acute endoplasmic reticulum stress (ERS) induces liver injury through the extensive and preferential decrease in both nuclear and chromatin-bound levels of HNF4α, and the inhibition of SRC kinase prevents HNF4α degradation following acute ERS [92]. Markedly, the recruitment of HNF4α in hepatocyte development is interconnected with other genes, which are the major contributors to the proper maintenance of hepatocyte-specific genes. Liver receptor homolog-1 (LRH1) overexpression protects hepatocytes from acute acetaminophen intoxication or hepatitis B virus (HBV)-induced acute liver failure. The increased expression of HNF4α maintains the hepatocyte identity and normal liver function, as it is under the control of LRH1 [93]. Another study reported the NRF2–HNF4α regulatory axis to be a cytoprotective response during hepatitis C virus (HCV) infection [94]. During HCV infection, NRF2 is activated, and it reduces HNF4α expression by altering the stability or decreasing the transcription of HNF4α [94,95]. HCV infection also promotes STAT3 expression through NRF2-ARE [94]. This STAT3 expression is negatively correlated with HNF4α, and stress-induced activation of the STAT3–HNF4α inflammatory loop leads to the decreased expression of miR-122, which is responsible for liver cancer cell migration and invasion [94,96].

In PiZ mouse (a model for this disease) livers, it was found that the downregulation of HNF4α was associated with the reduced expression of CEBPA, HNF1α, and NRF2 at an early age of the mice [97]. Reduced HNF4α was associated with the increased activation of β-catenin, which is responsible for impaired liver zonation in livers expressing α1-antitrypsin variant Z (ATZ). Livers of AAT-deficient patients were found to have a severe perturbation of liver zonation and an increased risk of HCC [97]. In DEN-induced HCC rats, it was found that HNF4α was significantly downregulated with the reduction of E-cadherin, whereas the expression of vimentin was notably increased [98]. In the same study, the overexpression of HNF4α blocked hepatocyte EMT in DEN-induced hepatocarcinogenesis. The explanation for the observed reduction in tumorigenesis was the suppression of β-catenin nuclear translocation and transcriptional activity by HNF4α in hepatocytes [98]. Moreover, liver-specific knockout of HNF4α mice revealed significantly enlarged livers with higher body weight; this correlated with the increased serum bile acid concentrations and defective lipid metabolism [74]. It has been reported that the acute loss of hepatic HNF4α causes hypotriglyceridemia, hypocholesterolemia, and fatty liver via reducing VLDL secretion and de novo cholesterol biosynthesis. A recent study by Huang’s group found that, in an NAFLD rat model, the activation of the transcriptional network of HNF4α with HNF4α-specific small activating RNA (saRNA) reduces the liver triglyceride, increases the high-density lipoprotein and low-density lipoprotein ratio, and decreases the white adipose tissue and body weight ratio [99]. Taken together, these studies provided evidence that HNF4α is important for lipid metabolism.

In a case study of diazoxide-responsive hyperinsulinism (HI) in children, it was found that HNF1α and HNF4α mutations accounted for 5.9% of all cases (12/204) [100]. Moreover, HNF1A-mutated hepatocellular adenomas (H-HCAs) were phenotypically categorized by marked steatosis [37] in which the mTOR pathway was aberrantly activated. Furthermore, these tumors exhibited an increase of eIF-4G3 and eEF1A2, at both mRNA and protein levels, which are involved in protein translation. This study also confirmed that cyclin D1 and eIF-4G3 are closely correlated with HNF1α expression at the protein level [37]. Another study reported that HNF1α inhibition in liver cancer cell lines caused the loss of cell–cell contacts and the development of migration structures with the upregulation of TGFB1, SNAIL, and SLUG, suggesting EMT is triggered by the loss of HNF1α expression [60]. In human liver cancers, PPARG expression is significantly increased by the activation of the Akt signaling responsible for liver pathology. The loss of function of HNF1α in liver lesions induces PPARG expression, resulting in tumorigenesis [36].

### 2.6. Disruptions of Epigenetic and miRNA Controls in HNF4α and HNF1α Networks Contribute to Cancer Development in Liver Cancer

While the role of mutations is well established in the development of cancer, disruptions of the transcription network are not limited to the changes in DNA sequence. Epigenetic alterations in healthy cells are important mechanisms for regulation of gene expression allowing, e.g., tissue differentiation [101]. The molecular basis encompasses post-translational histone modifications, which can influence heterochromatin and euchromatin changes; methylation of cytosine directly in the DNA molecule, as well as a plethora of nonstructural changes such as involvement of non-coding RNAs.

Protein arginine methyl transferase 1 (PRMT1) regulates the transcription of HNF4α by arginine methylation at HNF4α promoter. When PRMT1 is absent, demethylase, JMJD6 demethylates the HNF4α promoter and represses its expression [102]. Interestingly, a lack of PRMT1 causes hepatocyte proliferation, and the function of PRMT1 is strongly inhibited by alcohol, suggesting that the PRMT1–HNF4α axis plays a significant role in alcohol-related liver cancer development. On the other hand, inhibition of PRMT5 is known to cause induction of HNF4α by inhibiting histone H4 arginine-3 symmetrical dimethylation of HNF4 α promoter. Notably, a PRMT5 inhibitor, DW14800, reduces the proliferation and migration of HCC cells [103]. Modification of the epigenetic status of liver cancer cell line HepG2 by 5-Azacytidine (5-AZA) and Vitamin C led to increased levels of HNF4α along with elevated drug metabolic capacity [104]. Moreover, 5-AZA treatment increased E-cadherin and decreased SNAIL expression, suggesting that this treatment inhibits EMT through the induction of HNF4α expression. Additionally, HNF4α is also known to regulate ten-eleven translocation methylcytosine dioxygenase 3 (TET3), a protein whose function is tightly connected with DNA demethylation through its enhancer region [105]. Additionally, TET3 interacts with HNF4α and maintains both acetylation of lysine 27 of histone 3 (H3K27ac) and 5-hydroxymethylcytosine (5hmc) [105], which, in turn, controls the active epigenetic state of enhancers that induce transcription of genes in hepatocytes.

Among non-coding RNAs associated with cancer, of particular attention is miR-122, the most frequently detected miRNA in the liver and a direct target of HNF4α [106]. miR-122 is capable of inhibiting HCC progression by blocking the ADAM17 signal pathway [107]. The increased expression of miR-122 negatively regulates EMT and the metastasis of HCC cells through suppression of RhoA in the RhoA/Rock pathway, thereby increasing cell adhesion, cell junctions, and decreasing cell motility [108]. The HNF4α/miR-122 axis comes into play in HBV-associated HCC as well, where a ChIP assay showed a decreased association of HNF4α with miR-122 promoter in HBV-infected hepatoma cells [109]. The decreased expression of miR-122 in clinical samples of HCC correlated with grave prognosis and tumor progression [110]. miR-194/192 is also known to be regulated by HNF4α in hepatic cells [59]. miR-194/192 targets genes are responsible for glucose metabolism (*Gyg1),* EMT *(Msn),* tumor growth *(Rap2b* and *Ereg),* as well as cell adhesion *(Alcam)* [59]. Additionally, the HNF4α–miR-134 axis downregulates oncoprotein KRAS in HCC [111], whereas HNF4α-induced miR-124 and miR-7 repress RelA, a subunit of the NF-κB family (p65) and consequently diminish NF-κB activation [112] (Figure 1). In the feedback loop NF-κB upregulates the expression of miR-21, which reduces the level of HNF4α. miR-34a is upregulated by β-catenin, often mutated in HCC and targets HNF4α. Inhibition of this microRNA exerts anti-proliferative effect in hepatocyte culture by changing the levels as well as transcriptional activity of HNF4α [113]. However, in neuroblastoma, a pediatric solid tumor miR-34a appears to play anti-oncogenic role, while also regulating levels of HNF4α [114]. HNF4α is also involved in the axis with miR-542-3p and lncRNA, SNHG16 in neuroblastoma [115].

Temporary deactivation of HNF4α triggers a feedback loop consisting of miR-124, IL6R, STAT3, miR-24, and miR-629, leading to carcinogenesis [25] (Figure 1). After activation, this loop was able to sustain the inhibition of HNF4α and carcinogenesis. HCV was also capable of perturbing the network of miR-24, miR-629, miR-124 and HNF4α [95]. Systemic administration of miR-124 was also able to stop HCC growth in DEN-treated mice [25].

In pediatric and adolescent acute myeloid leukemia (AML) lncRNA, LINP1 was found to be overexpressed at diagnosis and reduced after complete remission. Furthermore, its knockdown was able to suppress glucose metabolism by downregulating HNF4α expression [116]. Another crucial cellular event controlled by HNF4α-non-coding RNAs axis is the regulation of EMT. Apart from the abovementioned examples, HNF4α also induces the expression of miR-29a and -29b, which in turn reduces the expression of DNA methyltransferase 3 (DNMT3) enzyme, playing an important role in EMT [117]. HNF4α is also able to repress transcription of another lncRNA, HOTAIR, by changing the chromatin structure. HOTAIR conveys SNAIL-mediated repression of epithelial genes [118,119].

**Figure 4:**
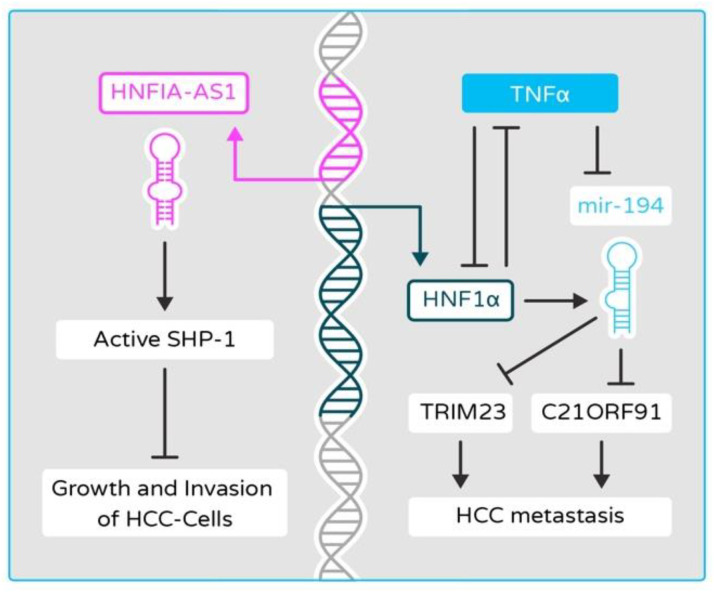
HNF1α/HNF1A-AS1/miR-194 pathway in epithelial–mesenchymal transition (EMT) and hepatocellular carcinoma (HCC) metastasis. HNF1α increases the activity of Src homology 2 domain-containing protein tyrosine phosphatase 1 (SHP-1) in human HCC cells via upregulating HNF1α antisense RNA 1 (HNF1A-AS1) and inhibits hepatic carcinogenesis. HNF1α induces the expression of miR-194, which inhibits the TNFα/NF-kB pathway and its downstream targets (tripartite motif containing 23, TRIM23, and Chromosome 21 Open Reading Frame 91, C21ORF91), and thus inhibits HCC metastasis. TNFα reciprocally inhibits the abundance of miR-194 by suppressing the expression of HNF1α in HCC cells and induces the expression of TRIM23 and C21ORF91, which play a role in HCC metastasis.

HNF1α antisense RNA 1 (HNF1A-AS1) is an lncRNA directly activated by HNF1α. This lncRNA was able to stop the growth and invasion of HCC cells via increasing the phosphatase activity of Src homology 2 domain-containing protein tyrosine phosphatase 1 (SHP-1) [120] (Figure 4). In HCC samples carrying HNF1α mutations, downregulation of miR-107 was found and hypothesized to contribute to lipid accumulation in HNF1α-deficient cells [121]. A low level of miR-194 is known to be correlated to vascular invasion in HCC with TRIM23 (tripartite motif containing 23), a ligase that is important for NF-κB activation, being the target of miR-194 (Figure 4). In turn, TNFα, which activates the NF-κB pathway, negatively regulates the expression of miR-194 via suppression of HNF1α [52]. Forced expression of HNF1α in HCC cells using a recombinant adenovirus upregulated the levels of miR-192 and -194 [122]. On the other hand, miR-192 and -194 levels were also significantly reduced in mice in which HNF1α was genetically deleted [123]. miR-194 was revealed to target human FZD6, while there exists an inverse correlation of miR-194 and Fzd6 in the mouse model of HCC.

### 2.7. Function of HNF4α and HNF1α Is Disrupted by HBV and HCV Infection

Hepatotropic viruses are a heterogenous group consisting of different genera and connected by their primary infection site. The two most prominent ones, HBV and HCV, are, respectively, a partially double-stranded DNA virus belonging to the Hepadnaviridae family and a single-stranded RNA virus of the Flaviviridae family. Both of them are directly connected to HCC, with chronic HBV infection accounting for at least 50% of cases worldwide and HCV being the main etiological factor in low-incidence areas [124]. Both viruses also interact with HNF4α and HNF1α TFs and the nature of this interaction appears to be bidirectional. The expression of HNF4α is suppressed in HCC exhibiting long-term expression of HBV with a slight reduction in the level of HNF1α and yet HBV core-related antigen (HBcrAg) appears to upregulate the transcription of HNF4α [109,125,126,127]. Interestingly, suppression is not present during transient infection. The HBx protein, a multifunctional protein encoded by the HBV genome, was found to downregulate HNF4α at the transcriptional level through the activation of ERK signaling pathway [127]. Furthermore, HBV-induced HNF4α suppression appears to increase cell proliferation in vitro and contribute to tumor development in nude mouse xenograft models [127]. On the other hand, HNF4α seems to be positively linked with higher HBV replication [128,129,130,131,132]. Conversely, the level of HNF4α was significantly elevated in the liver biopsies of patients with severe hepatitis B compared to patients with chronic hepatitis B and liver cirrhosis in the course of HBV infection [133]. Unfortunately, none of the clinical studies include an uninfected control group, which prevents comparing HNF4α levels between long-term HBV infection and healthy tissue. Moreover, several factors, such as TRAIL, luteolin, epigallocatechin gallate, and p22-FLIP, which influence HBV replication, seem to exert their effects through targeting HNF4α [134,135,136,137,138]. HNF1α’s influence on HBV replication is less clear with existing contradictory research. HNF1α is capable of downregulating HBV replication and antigen expression [139]; however, because of its connection with HNF4α, it can yield the opposite effect as well [140,141,142].

HCV infection is known to suppress the expression of HNF4α, correlating with decreased protein levels of microsomal triglyceride transfer protein (MTP) and HNF1α [95]. Furthermore, infection with HCV led to loss of HNF4α by inducing the upregulation of miR-24 and miR-629 with concurrent downregulation of miR-124 expression levels [95]. This effect appears to be mediated through HCV-derived small non-coding RNAs, Vmr11. Additionally, the loss of HNF4α in liver tumors of HCV-infected humanized mice was correlated with the induction of EMT-related genes [143]. However, it was found that HCV-induced upregulation of glycolysis was controlled by the activation of HNF4α [144]. HNF4α is also important for the successful completion of the HCV viral cycle [145]. During infection, the expression of HNF1α is significantly repressed, resulting in an increase of serum lipid uptake by the liver [146]. This appears to be at least mediated by lysosomal degradation via chaperone-mediated autophagy promoted by the NS5A protein [147,148,149]. In summary, while HBV infection appears to decrease the levels of HNF4α in long-term infection, HNF4α itself is important for HBV replication. This phenomenon may be related to differences between severe hepatitis, in which HBV replication is high, and chronic hepatitis leading to HCC, in which HBV replication occurs at a low level. The research relating to HNF1α is, however, contradictory, and it is difficult to summarize its role in HBV infection. A preponderance of evidence suggests that HCV infection suppresses the expression of both HNF4α and HNF1α, while, as in the case of HBV, HNF4α is essential for HCV replication. Several papers have mentioned the possibility of anti-HBV and anti-HCV drugs that influence HNF4α levels; however, their use may lead to worse clinical outcomes, as HNF4α is an important factor in HCC.

### 2.8. HNF4α in Colorectal Cancer (CRC): Friend or Foe?

Despite ample evidence suggesting HNF4α acts as a tumor suppressor in liver cancer, its role in other types of cancers is still unclear. Aberrations in the HNF4α signaling pathway have been reported in many GIT cancers including colon cancer, gastric cancer, and pancreatic cancer [150,151,152,153,154,155,156,157]. Nevertheless, most of these reports with respect to the role of HNF4α in colon cancer are still unclear. Many studies have ascribed a tumor suppressor role to HNF4α in CRC [22,158,159,160]. However, other published reports have also suggested the tumor promoter effects of HNF4α, depending on the tissue and the isoform of HNF4α expressed in the specific tissue [161,162,163,164,165]. The chromosome 20q amplification is one of the main genomic alterations in CRC identified by TCGA, and HNF4α is the candidate driver gene for this amplification [163,166,167]. Proteomic characterization of CRC established HNF4α as one of the top genes whose amplification is highly correlated with its protein levels and suggested an oncogenic role for HNF4α [163]. Data from the Achilles project established that HNF4α shRNA knockdown has a negative effect on proliferation and viability of colon cancer cells [163,168]. The limitation of these studies is that they did not determine the isoform of HNF4α. Nonetheless, a preponderance of evidence points to the fact that the role of HNF4α in cancer is not only isoform-specific but also depends on the status of HNF4α gene amplification. Schwartz et al. proposed an oncogenic role for HNF4α in CRC by combining the use of HNF4α antagonists of the MEDICA family and siRNA-mediated inhibition of HNF4α in CRC cell lines [161]. Furthermore, they demonstrated that the tumor-promoting effect of HNF4α is due to its interaction with anti-apoptotic oncogenes and cytokines. MEDICA analogues inhibited the proliferation of CRC cell lines evidenced by increased cell apoptosis, caspase-3 activity, cytosolic cytochrome-c, and PCNA. The inhibition of cell growth and HNF4α expression by MEDICA analogues were compared to troglitazone, a known CRC suppressor [161]. In an adenomatous polyposis coli (Apc^Min^) mouse model, deletion of both P1 and P2-HNF4α inhibited the initiation of intestinal polyposis and neoplasia. The possible explanation for the tumor-promoting effect of HNF4α is that the decreased production of ROS and ROS-triggered apoptosis in cancerous cells favors cancer cell survival and proliferation [162].

Long intergenic 20 non-coding RNA 00511 (LINC00511) is highly expressed in CRC tumors and CRC cell lines, and silencing LINC00511 has anti-proliferative and proapoptotic action on CRC cell lines. LINC00511 is under the transcriptional control of HNF4α in CRC. HNF4α also inhibits IL-24, a proinflammatory and tumor suppressive pathway, through upregulation of LINC00511 and promotes CRC proliferation, migration, and metastasis [169]. The low level of another lncRNA, LINC00483, was found to be correlated with harmful clinical features of colorectal cancer [170]. In vitro experiments showed a likely role of LINC00483 in the inhibition of EMT through a negative regulation by HNF4α. Xu et al. reported that lncRNA LINC00858 is highly expressed in colon cancer tissue compared to normal adjustment tissue and correlated with poor differentiation, higher TNM stages, and metastasis to lymph nodes [171]. The knockdown of LINC00858 reduced the invasion and migration capacities of colon cancer cell lines, whereas its overexpression did the opposite. All of the above is possibly mediated through HNF4α, as LINC00858 was found to bind to and increase the expression of HNF4α. Interestingly, upregulation of the LINC00858/HNF4α axis, in turn, leads to downregulation of WNK2, which acts as tumor-promoting mechanism [171].

Both P1 and P2 isoforms of HNF4A are expressed in the small and large intestine, and many studies have suggested a tumor suppressant role for P1-HNF4α and an oncogenic role for P2-HNF4α in CRC. The downregulated expression of P1-HNF4α isoform in CRC was proposed because of the SRC tyrosine kinase-dependent phosphorylation of P1-HNF4α, which leads to increased instability and degradation of HNF4α [150,153,172]. In a mouse CRC xenograft model, the ectopic expression of P1-HNF4α inhibited tumor growth by competing with Wnt/β-catenin mediator TCF4 for binding to the target gene promoters and led to inhibition of Wnt/β-catenin signaling [173]. A recent study by Babeu et al. established that P1-HNF4α isoforms predominantly play a role in colonic cell differentiation and cell metabolism and have an anti-tumorigenic function, while P2-HNF4α isoforms are the main drivers of colonic cell proliferation and have an oncogenic role in CRC [174]. Activating mutations of the Wnt/β-catenin pathway are well established in CRC and are implicated in specifically repressing the expression of P1-HNF4α in CRC and thus favor tumor development. How β-catenin selectively inhibits the P1-HNF4α gene transcripts is yet to be elucidated. Altogether, the mRNA and protein expression of P1-HNF4α isoforms is inhibited in CRC in comparison to P2-HNF4α isoforms whose expression is maintained or increased in the CRC tumors [150,153,174]. The downregulated expression of the P1-HNF4α isoform is suggested as a marker for worse prognosis and liver metastasis in CRC patients [152]. The role of HNF4α in gastric and pancreatic cancer is beyond the scope of this review and the reader is referred to a recent review by Lv et al. [175]. In summary, the role of HNF4α in CRC is complex, depending on the isoform expressed, the tumor microenvironment, and the underlying genomic aberrations. Thus, whether HNF4α is a friend or a foe in CRC is puzzling, and future studies are needed to elucidate its role in cancer and to make it an actionable target for cancer treatment.

## 3. HNF4α as a Therapeutic Target in Cancer

The emerging role of HNFs in cellular proliferation, differentiation, metabolism, and immunity has highlighted their therapeutic potential. HNF4α was originally described as an orphan nuclear receptor continuously bound to fatty acids. Linoleic acid has been proposed as a reversible ligand for HNF4α expression in the mouse liver [176]. Studies are underway to examine HNF4α as a therapeutic target, and recently, the alkaloid Berberine was found to upregulate the expression of HNF4α [177]. By developing highly specific small molecules that can selectively target the diseased cell, HNF4α will offer new therapeutic options for cancer treatment [178]. Recently, mesenchymal stem cells (MSCs) have emerged as efficient vehicles for tumor-targeted gene therapy owing to their openness to genetic manipulation and homing capacity toward the tumor site [179]. MSC-based gene delivery of HNF4α could be a propitious avenue for HCC prevention [180]. Cancer gene therapy is gaining ground, and TFs can serve as promising candidates for delivery into cancer cells by acting as master regulators and exerting control over a wide set of genes and cellular processes [181]. Conjunctional transduction of HNF4α, HNF1α, and FOXA3 in HCC cell lines induces their differentiation into mature hepatocytes, and the transduced cells lose tumorigenicity in vivo [182]. Therefore, identifying pathogenic mutations of HNF1α and HNF4α has implications for liver cancer biology and drug targets for precision medicine. Indeed, HNF1α was proposed to serve as a personalized candidate mutation-driver gene in HCC [183]. As such, the multi-omics methodology can be used to identify candidate mutations and develop therapeutic targets.

## 4. Conclusions

HNF4α and HNF1α are known to be master regulators of the development of the liver. As a matter of fact, their loss of function causes severe damage in liver formation. However, the roles of these factors in liver cancer development and progression have been demonstrated only recently. In this review, we summarized the possible mechanisms associated with HNF4α and HNF1α that regulate multiple oncogenic pathways in the liver and discussed the potential use of HNF4α as a therapeutic target for liver cancer. Therefore, identifying a link between their loss-of-function mutations and the molecular mechanism for tumorigenesis will help to improve understanding of how HNF4α and HNF1α function in liver cancer.
